# (2*R*,4*R*)-2-Hydr­oxy-4-(2-methoxy­phen­yl)bicyclo­[3.3.1]nonan-9-one

**DOI:** 10.1107/S1600536809028980

**Published:** 2009-07-29

**Authors:** Shuping Luo, Guangcun Zhang, Shuai Zhang, Yifeng Wang, Wei Zhang

**Affiliations:** a State Key Laboratory Breeding Base of Green Chemistry-Synthesis Technology, Zhejiang University of Technology, Hangzhou 310014, People’s Republic of China

## Abstract

The title compound, C_16_H_20_O_3_, contains a bicyclic ring system with two chiral centers. The crystal structure is stabilized by inter­molecular O—H⋯O hydrogen bonds. The absolute configuration was established by the stereo-selectivity of the asymmetric organocatalysis.

## Related literature

A similar structure is described by Cao *et al.* (2007 ▶). For general background to organocatalysis, see: List *et al.* (2000 ▶, 2001 ▶); Notz *et al.* (2001 ▶).
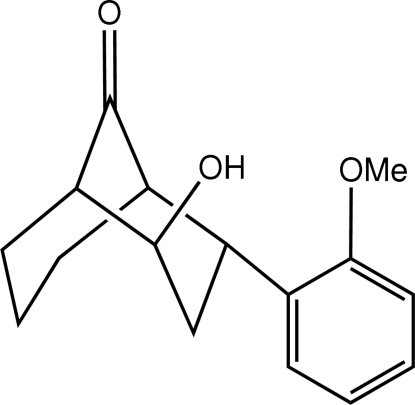


## Experimental

### 

#### Crystal data

C_16_H_20_O_3_
*M* *_r_* = 260.33
Orthorhombic, 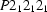

*a* = 6.9378 (5) Å
*b* = 12.5291 (11) Å
*c* = 16.0726 (14) Å
*V* = 1397.1 (2) Å^3^
*Z* = 4
Mo *K*α radiation
μ = 0.08 mm^−1^
*T* = 296 K
0.47 × 0.32 × 0.29 mm

#### Data collection

Rigaku R-AXIS RAPID diffractometer
Absorption correction: multi-scan (*ABSCOR*; Higashi, 1995 ▶) *T*_min_ = 0.945, *T*_max_ = 0.976
13314 measured reflections
1840 independent reflections
1211 reflections with *F* ^2^ > 2σ(*F* ^2^)
*R*_int_ = 0.034

#### Refinement

*R*[*F* ^2^ > 2σ(*F* ^2^)] = 0.032
*wR*(*F* ^2^) = 0.061
*S* = 1.00
1840 reflections
173 parameters
H-atom parameters constrained
Δρ_max_ = 0.16 e Å^−3^
Δρ_min_ = −0.13 e Å^−3^

### 

Data collection: *PROCESS-AUTO* (Rigaku, 2006 ▶); cell refinement: *PROCESS-AUTO*; data reduction: *CrystalStructure* (Rigaku/MSC, 2004 ▶), and Larson (1970 ▶); program(s) used to solve structure: *SIR97* (Altomare *et al.*, 1999 ▶); program(s) used to refine structure: *CRYSTALS* (Watkin *et al.*, 1996 ▶); molecular graphics: *ORTEP-3 for Windows* (Farrugia, 1997 ▶); software used to prepare material for publication: *CrystalStructure* (Rigaku/MSC, 2004 ▶).

## Supplementary Material

Crystal structure: contains datablocks global, I. DOI: 10.1107/S1600536809028980/zl2227sup1.cif

Structure factors: contains datablocks I. DOI: 10.1107/S1600536809028980/zl2227Isup2.hkl

Additional supplementary materials:  crystallographic information; 3D view; checkCIF report

## Figures and Tables

**Table 1:** Hydrogen-bond geometry (Å, °)

*D*—H⋯*A*	*D*—H	H⋯*A*	*D*⋯*A*	*D*—H⋯*A*
O1—H101⋯O2^i^	0.85	1.99	2.8268 (19)	171
O1^ii^—H101^ii^⋯O2	0.85	1.99	2.8268 (19)	171
C14—H14⋯O1^iii^	0.93	2.51	3.434 (2)	176

Symmetry codes: (i) 
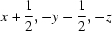
; (ii) 
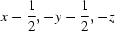
; (iii) 
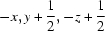
.

**Fig. 1.**
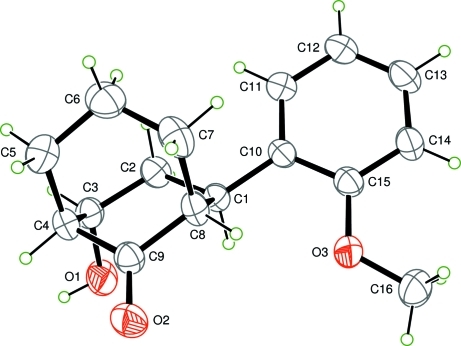
Thermal ellipsoid representation of the of title compound with the atomic labeling scheme. Displacement ellipsoids are drawn at the 40% probability level.

**Fig. 2.**
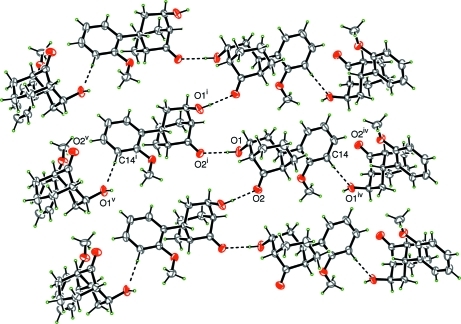
View of the hydrogen bonding interactions. H bonds are represented as dashed lines. Symmetery codes: (i) x + 1/2, -y - 1/2, -z. (iv) -x, y + 1/2, 1/2 -z. (v) 1/2 - x, -y - 1, - 1/2 + z.

## supplementary crystallographic information

### Comment

The natural amino acid *L*-proline, pioneered by List and Barbas III and their co-workers, has been fully appreciated as an attractive enantioselective organocatalyst for direct asymmetric carbon-carbon and carbon-heteroatom bond-forming reactions, such as aldol (List *et al.*, 2000), Mannich (List *et al.*, 2001) and Michael (Notz *et al.*, 2000) reactions. In our laboratory, a novel tandem Michael-aldol reaction of ketones with cinnamaldehyde derivatives catalyzed by *L*-proline was developed and a series of new products was obtained. The crystal structure of one of these, the title compound, is reported in this article.

In the crystal structure of the title compound (Fig. 1), both bicyclic six-membered rings display chair conformations in which atoms C1, C8, C4, C3 and atoms C4, C8, C7, C5 each lie in an approximate plane with the dihedral angle between them being 115.9 (0)°. C9 is located above the two planes with similar dihedral angles. The hydroxyl group and the phenyl group are located on different sides of the plane made up of atoms C1, C8, C4, C3. The hydroxyl group is in an axial position, the phenyl group in an equatorial position of the cyclohexanone ring.

Intermolecular O—H···O hydrogen bonds (Tab. 1) connect neighboring molecules with each other to form a one-dimensional chain that stretches along the direction of the a axis (Fig. 2). Via weak C—H···O hydrogen bonds (Tab. 1) molecules are linked along the b axis to form another one-dimensional chain.

### Experimental

A DMF (2 ml) solution of cyclohexanone and 3-(2-methoxyphenyl)acryaldehyde in the presence of *L*-proline as organocatalyst was stirred at room temperature for 48 h. Then the mixture was washed with water (20 ml) and extracted with ethyl acetate (three times). The organic solvent was removed under reduced pressure and the product was purified by silica gel chromatography (pentane: ethyl acetate mixtures). Suitable crystals were obtained by slow evaporation of ethanol at room temperature.

### Refinement

In the absence of significant anomalous scatterers Friedel pairs were merged prior to refinement. All H atoms were placed in calculated positions with C—H = 0.98 Å (*sp*), C—H = 0.97 Å (sp2), C—H= 0.96 Å (sp3), C—H = 0.93 Å (aromatic) and O—H = 0.85 Å and included in the final cycles of refinement in a riding motion approximation, with *U*_iso_(H) = 1.2*U*_eq_ of the carrier atoms.

### Crystal data

**Table:**

C_16_H_20_O_3_	*F*(000) = 560.00
*M**_r_* = 260.33	*D*_x_ = 1.238 Mg m^−^^3^
Orthorhombic, *P*2_1_2_1_2_1_	Mo *K*α radiation, λ = 0.71075 Å
Hall symbol: P 2ac 2ab	Cell parameters from 8871 reflections
*a* = 6.9378 (5) Å	θ = 3.0–27.4°
*b* = 12.5291 (11) Å	µ = 0.08 mm^−^^1^
*c* = 16.0726 (14) Å	*T* = 296 K
*V* = 1397.1 (2) Å^3^	Chunk, colorless
*Z* = 4	0.47 × 0.32 × 0.29 mm

### Data collection

**Table:**

Rigaku R-AXIS RAPID diffractometer	1211 reflections with *F*^2^ > 2σ(*F*^2^)
Detector resolution: 10.00 pixels mm^-1^	*R*_int_ = 0.034
ω scans	θ_max_ = 27.4°
Absorption correction: multi-scan (*ABSCOR*; Higashi, 1995)	*h* = −8→8
*T*_min_ = 0.945, *T*_max_ = 0.976	*k* = −16→16
13314 measured reflections	*l* = −20→20
1840 independent reflections	

### Refinement

**Table:**

Refinement on *F*^2^	*w* = 1/[1.01σ(*F*_o_^2^)]/(4*F*_o_^2^)
*R*[*F*^2^ > 2σ(*F*^2^)] = 0.032	(Δ/σ)_max_ < 0.001
*wR*(*F*^2^) = 0.061	Δρ_max_ = 0.16 e Å^−^^3^
*S* = 1.00	Δρ_min_ = −0.13 e Å^−^^3^
1840 reflections	Extinction correction: Larson (1970), equation 22
173 parameters	Extinction coefficient: 649 (27)
H-atom parameters constrained	

### Special details

**Table:**

Geometry. ENTER SPECIAL DETAILS OF THE MOLECULAR GEOMETRY
Refinement. Refinement using all reflections. The weighted *R*-factor (*wR*) and goodness of fit (*S*) are based on *F*^2^. *R*-factor (gt) are based on *F*. The threshold expression of *F*^2^ > 2.0 σ(*F*^2^) is used only for calculating *R*-factor (gt).

### Fractional atomic coordinates and isotropic or equivalent isotropic displacement parameters (Å^2^)

**Table:**

	*x*	*y*	*z*	*U*_iso_*/*U*_eq_	
O1	0.3084 (2)	−0.19303 (10)	0.07830 (9)	0.0638 (4)	
O2	−0.1217 (2)	−0.10943 (12)	0.00114 (9)	0.0622 (4)	
O3	−0.10291 (19)	0.08144 (11)	0.23327 (8)	0.0593 (4)	
C1	0.1869 (2)	0.03037 (14)	0.12399 (11)	0.0390 (5)	
C2	0.3817 (2)	−0.00912 (14)	0.09036 (12)	0.0457 (5)	
C3	0.3617 (2)	−0.10201 (16)	0.02981 (12)	0.0476 (5)	
C4	0.2100 (2)	−0.08335 (16)	−0.03793 (12)	0.0472 (5)	
C5	0.2559 (3)	0.00623 (18)	−0.10098 (12)	0.0601 (6)	
C6	0.2481 (3)	0.11937 (17)	−0.06523 (13)	0.0657 (7)	
C7	0.0787 (3)	0.13738 (16)	−0.00841 (12)	0.0599 (6)	
C8	0.0362 (2)	0.04651 (14)	0.05297 (12)	0.0433 (5)	
C9	0.0240 (2)	−0.05569 (16)	0.00463 (12)	0.0440 (5)	
C10	0.1996 (2)	0.12898 (14)	0.17882 (11)	0.0388 (5)	
C11	0.3544 (2)	0.19787 (16)	0.17853 (12)	0.0519 (6)	
C12	0.3625 (3)	0.28667 (17)	0.23012 (12)	0.0653 (7)	
C13	0.2124 (3)	0.30819 (18)	0.28234 (13)	0.0632 (7)	
C14	0.0528 (3)	0.24199 (16)	0.28470 (12)	0.0527 (6)	
C15	0.0475 (2)	0.15310 (14)	0.23345 (12)	0.0437 (5)	
C16	−0.2463 (2)	0.0914 (2)	0.29575 (13)	0.0707 (7)	
H1	0.1366	−0.0270	0.1593	0.047*	
H3	0.4868	−0.1155	0.0035	0.057*	
H4	0.1918	−0.1502	−0.0687	0.057*	
H8	−0.0897	0.0599	0.0785	0.052*	
H11	0.4567	0.1845	0.1426	0.062*	
H12	0.4697	0.3312	0.2291	0.078*	
H13	0.2174	0.3679	0.3167	0.076*	
H14	−0.0495	0.2570	0.3202	0.063*	
H21	0.4604	−0.0320	0.1369	0.055*	
H22	0.4448	0.0495	0.0619	0.055*	
H51	0.3847	−0.0058	−0.1226	0.072*	
H52	0.1634	0.0017	−0.1461	0.072*	
H61	0.3655	0.1322	−0.0341	0.079*	
H62	0.2399	0.1696	−0.1110	0.079*	
H71	−0.0348	0.1477	−0.0427	0.072*	
H72	0.1032	0.2016	0.0236	0.072*	
H101	0.3263	−0.2488	0.0498	0.077*	
H161	−0.3243	0.0282	0.2967	0.085*	
H162	−0.1856	0.1007	0.3489	0.085*	
H163	−0.3260	0.1522	0.2839	0.085*	

### Atomic displacement parameters (Å^2^)

**Table:**

	*U*^11^	*U*^22^	*U*^33^	*U*^12^	*U*^13^	*U*^23^
O1	0.0884 (9)	0.0367 (7)	0.0664 (9)	0.0155 (8)	0.0188 (8)	0.0031 (7)
O2	0.0536 (7)	0.0633 (10)	0.0698 (10)	−0.0144 (8)	−0.0018 (8)	−0.0159 (9)
O3	0.0580 (7)	0.0607 (9)	0.0591 (9)	−0.0141 (8)	0.0173 (7)	−0.0219 (7)
C1	0.0433 (9)	0.0327 (10)	0.0410 (10)	0.0023 (8)	0.0029 (8)	−0.0021 (8)
C2	0.0448 (10)	0.0422 (11)	0.0502 (12)	0.0061 (10)	−0.0038 (9)	−0.0003 (10)
C3	0.0494 (11)	0.0398 (11)	0.0537 (12)	0.0076 (10)	0.0110 (10)	−0.0023 (10)
C4	0.0553 (11)	0.0392 (11)	0.0472 (11)	−0.0009 (10)	0.0070 (10)	−0.0121 (9)
C5	0.0638 (13)	0.0677 (14)	0.0488 (12)	0.0024 (13)	0.0070 (11)	0.0001 (11)
C6	0.0879 (16)	0.0525 (14)	0.0566 (14)	−0.0027 (14)	−0.0052 (13)	0.0114 (12)
C7	0.0857 (15)	0.0436 (12)	0.0503 (12)	0.0147 (12)	−0.0154 (12)	−0.0024 (11)
C8	0.0421 (9)	0.0428 (11)	0.0452 (10)	0.0068 (9)	−0.0030 (9)	−0.0122 (10)
C9	0.0454 (10)	0.0447 (11)	0.0420 (11)	−0.0015 (10)	−0.0025 (9)	−0.0042 (10)
C10	0.0491 (10)	0.0333 (10)	0.0341 (9)	0.0000 (9)	−0.0037 (9)	−0.0003 (8)
C11	0.0595 (12)	0.0452 (11)	0.0509 (12)	−0.0091 (11)	0.0081 (10)	−0.0048 (10)
C12	0.0772 (14)	0.0512 (14)	0.0675 (15)	−0.0229 (13)	0.0124 (13)	−0.0159 (12)
C13	0.0856 (15)	0.0440 (13)	0.0600 (14)	−0.0115 (13)	0.0037 (13)	−0.0156 (11)
C14	0.0650 (13)	0.0442 (12)	0.0488 (12)	0.0023 (11)	0.0065 (11)	−0.0104 (10)
C15	0.0519 (11)	0.0396 (11)	0.0397 (11)	−0.0022 (9)	−0.0024 (10)	−0.0023 (10)
C16	0.0503 (12)	0.0801 (17)	0.0816 (16)	−0.0057 (13)	0.0152 (12)	−0.0221 (14)

### Geometric parameters (Å, °)

**Table:**

O1—C3	1.430 (2)	O1—H101	0.846
O2—C9	1.216 (2)	C1—H1	0.980
O3—C15	1.376 (2)	C2—H21	0.970
O3—C16	1.419 (2)	C2—H22	0.970
C1—C2	1.537 (2)	C3—H3	0.980
C1—C8	1.561 (2)	C4—H4	0.980
C1—C10	1.520 (2)	C5—H51	0.970
C2—C3	1.523 (2)	C5—H52	0.970
C3—C4	1.533 (2)	C6—H61	0.970
C4—C5	1.545 (2)	C6—H62	0.970
C4—C9	1.501 (2)	C7—H71	0.970
C5—C6	1.531 (3)	C7—H72	0.970
C6—C7	1.505 (3)	C8—H8	0.980
C7—C8	1.535 (2)	C11—H11	0.930
C8—C9	1.500 (2)	C12—H12	0.930
C10—C11	1.378 (2)	C13—H13	0.930
C10—C15	1.406 (2)	C14—H14	0.930
C11—C12	1.389 (2)	C16—H161	0.960
C12—C13	1.365 (3)	C16—H162	0.960
C13—C14	1.384 (3)	C16—H163	0.960
C14—C15	1.386 (2)		
			
C15—O3—C16	118.18 (15)	O1—C3—H3	109.1
C2—C1—C8	111.91 (14)	C2—C3—H3	109.1
C2—C1—C10	114.46 (14)	C4—C3—H3	109.1
C8—C1—C10	110.96 (14)	C3—C4—H4	108.4
C1—C2—C3	112.99 (15)	C5—C4—H4	108.4
O1—C3—C2	106.53 (15)	C9—C4—H4	108.4
O1—C3—C4	109.33 (15)	C4—C5—H51	108.1
C2—C3—C4	113.56 (16)	C4—C5—H52	108.1
C3—C4—C5	115.78 (16)	C6—C5—H51	108.1
C3—C4—C9	107.58 (15)	C6—C5—H52	108.1
C5—C4—C9	107.97 (16)	H51—C5—H52	109.5
C4—C5—C6	114.78 (17)	C5—C6—H61	108.5
C5—C6—C7	113.23 (18)	C5—C6—H62	108.5
C6—C7—C8	115.36 (16)	C7—C6—H61	108.5
C1—C8—C7	115.97 (15)	C7—C6—H62	108.5
C1—C8—C9	107.82 (15)	H61—C6—H62	109.5
C7—C8—C9	108.12 (15)	C6—C7—H71	108.0
O2—C9—C4	124.46 (18)	C6—C7—H72	108.0
O2—C9—C8	122.92 (17)	C8—C7—H71	108.0
C4—C9—C8	112.62 (15)	C8—C7—H72	108.0
C1—C10—C11	123.56 (16)	H71—C7—H72	109.5
C1—C10—C15	119.53 (16)	C1—C8—H8	108.2
C11—C10—C15	116.91 (17)	C7—C8—H8	108.2
C10—C11—C12	122.11 (19)	C9—C8—H8	108.2
C11—C12—C13	119.6 (2)	C10—C11—H11	118.9
C12—C13—C14	120.6 (2)	C12—C11—H11	118.9
C13—C14—C15	119.12 (19)	C11—C12—H12	120.2
O3—C15—C10	115.32 (16)	C13—C12—H12	120.2
O3—C15—C14	123.07 (17)	C12—C13—H13	119.7
C10—C15—C14	121.60 (17)	C14—C13—H13	119.7
C3—O1—H101	109.0	C13—C14—H14	120.4
C2—C1—H1	106.3	C15—C14—H14	120.4
C8—C1—H1	106.3	O3—C16—H161	109.5
C10—C1—H1	106.3	O3—C16—H162	109.5
C1—C2—H21	108.6	O3—C16—H163	109.5
C1—C2—H22	108.6	H161—C16—H162	109.5
C3—C2—H21	108.6	H161—C16—H163	109.5
C3—C2—H22	108.6	H162—C16—H163	109.5
H21—C2—H22	109.5		
			
C16—O3—C15—C10	170.27 (16)	C5—C4—C9—C8	−62.1 (2)
C16—O3—C15—C14	−8.4 (2)	C9—C4—C5—C6	50.9 (2)
C2—C1—C8—C7	−67.5 (2)	C4—C5—C6—C7	−42.5 (2)
C2—C1—C8—C9	53.83 (19)	C5—C6—C7—C8	42.9 (2)
C8—C1—C2—C3	−47.7 (2)	C6—C7—C8—C1	69.4 (2)
C2—C1—C10—C11	19.8 (2)	C6—C7—C8—C9	−51.8 (2)
C2—C1—C10—C15	−160.11 (16)	C1—C8—C9—O2	116.16 (19)
C10—C1—C2—C3	−175.04 (15)	C1—C8—C9—C4	−63.67 (19)
C8—C1—C10—C11	−108.0 (2)	C7—C8—C9—O2	−117.7 (2)
C8—C1—C10—C15	72.1 (2)	C7—C8—C9—C4	62.4 (2)
C10—C1—C8—C7	61.7 (2)	C1—C10—C11—C12	−179.26 (18)
C10—C1—C8—C9	−176.97 (14)	C1—C10—C15—O3	1.2 (2)
C1—C2—C3—O1	−71.93 (19)	C1—C10—C15—C14	179.90 (17)
C1—C2—C3—C4	48.5 (2)	C11—C10—C15—O3	−178.77 (16)
O1—C3—C4—C5	−174.64 (15)	C11—C10—C15—C14	−0.0 (2)
O1—C3—C4—C9	64.54 (19)	C15—C10—C11—C12	0.7 (2)
C2—C3—C4—C5	66.5 (2)	C10—C11—C12—C13	−0.9 (3)
C2—C3—C4—C9	−54.3 (2)	C11—C12—C13—C14	0.4 (3)
C3—C4—C5—C6	−69.7 (2)	C12—C13—C14—C15	0.2 (3)
C3—C4—C9—O2	−116.3 (2)	C13—C14—C15—O3	178.22 (18)
C3—C4—C9—C8	63.5 (2)	C13—C14—C15—C10	−0.4 (2)
C5—C4—C9—O2	118.0 (2)		

### Hydrogen-bond geometry (Å, °)

**Table:**

*D*—H···*A*	*D*—H	H···*A*	*D*···*A*	*D*—H···*A*
O1—H101···O2^i^	0.85	1.99	2.8268 (19)	171
O1^ii^—H101^ii^···O2	0.85	1.99	2.8268 (19)	171
C14—H14···O1^iii^	0.93	2.51	3.434 (2)	176

Symmetry codes: (i) *x*+1/2, −*y*−1/2, −*z*; (ii) *x*−1/2, −*y*−1/2, −*z*; (iii) −*x*, *y*+1/2, −*z*+1/2.
